# Appendiceal fecalith misdiagnosed as cecal stromal tumor: don't fall into the trap

**DOI:** 10.1055/a-2333-9599

**Published:** 2024-07-29

**Authors:** Jinghao Li, Dan Liu, Deliang Li, Yangyang Zhou, Yue Zhao, Bingrong Liu

**Affiliations:** 1 191599 Department of Gastroenterology, The First Affiliated Hospital of Zhengzhou University, Zhengzhou, China

**Fig. 1:**
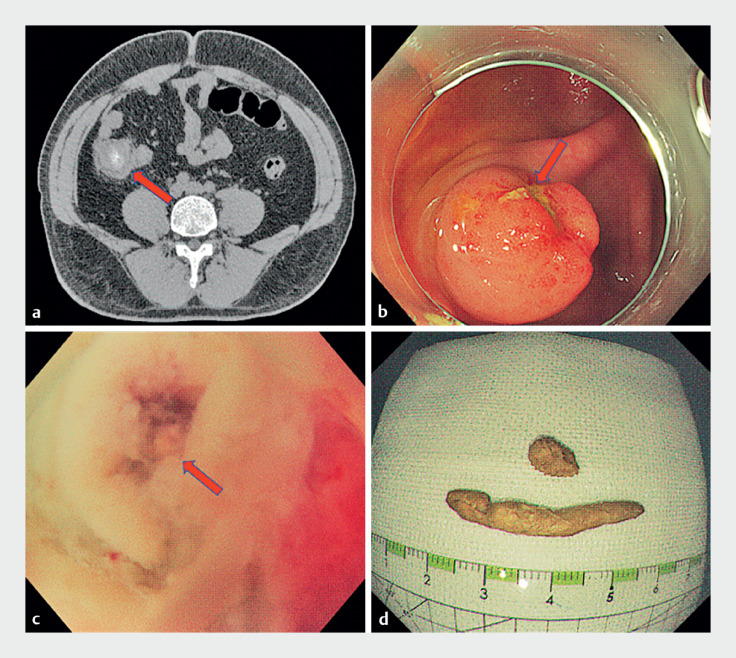
Imaging studies and the cause of symptoms. **a** Computed tomography of the abdomen showed a cecal mass (arrow) with central calcification. **b** Colonoscopy showed a mass of approximately 15×15 mm with superficial ulceration (arrow) in the cecum. **c** The appendix opening (arrow). **d** The fecaliths.

A 30-year-old man presented with a 2-day history of hematochezia and a 4-year history of intermittent abdominal pain. Computed tomography scan revealed a low-density mass with central calcification in the cecum ( Fig. 1 **a** ). Colonoscopy showed a 15×15 mm cecal mass with a superficial ulcer ( Fig. 1 **b** ). A cecal submucosal tumor was considered. The surgeon recommended ileocecectomy or partial cecectomy. The patient was afraid of surgery and was transferred to our department.

We performed a second colonoscopy, which revealed that the mass originated from the appendix opening and the “ulcer” appeared to be fecalith-like substance. We made a diagnosis of appendiceal fecalith, not submucosal tumor. A snare was applied to resect the fecalith. With the appendiceal orifice opened ( Fig. 1 **c** ), a fecalith stump was found in the appendiceal cavity, which confirmed our diagnosis.

Subsequently, endoscopic retrograde appendicitis therapy (ERAT) was performed ( Video 1 ). A catheter was inserted into the end of the appendiceal cavity, normal saline was used to flush the cavity, and two large fecaliths were flushed out ( Fig. 1 **d** ). Pathologic examination revealed inflammatory tissue. The patient recovered uneventfully and stayed stable during follow-up.

We demonstrate an interesting case of appendiceal fecaliths mimicking appendiceal tumor, which is likely to be misdiagnosed. We performed a minimally invasive procedure, endoscopic retrograde appendicitis therapy, to deal with it, which avoided traumatic surgery for the patient.Video 1

This case suggests that appendiceal fecalith causing mucosal inflammation may be misdiagnosed as cecal submucosal tumor. ERAT has both therapeutic and diagnostic value.

Endoscopy_UCTN_Code_TTT_1AQ_2AH

